# Health Tract No. 344

**Published:** 1868-11

**Authors:** 


HEALTH TRACT NO. 344.

### Marriage and Divorce.

It is stated that in a single year, there were sixteen hundred cases of divorce in the state of Massachusetts alone, a state which is perhaps farther advanced in general intelligence than any other in the Union. How many there are in Connecticut, the "Cutest State," where divorce is made easy, has not been made public; but, it is not denied that divorce is becoming more common in every Northern state than it was formerly, and very much more frequent than in the South. With these facts staring us in the face, a wail comes from every civilized land that marriages are becoming alarmingly infrequent, so much so in Massachusetts, that a society has been proposed for the purpose of encouraging marriages by endowing every marriage with a certain sum of money.

But both the pulpit and the press are uniting their forces to make a raid against a hidden crime on the part of the married, said to be becoming amazingly common—that of preventing the birth of children. Civil laws and regulations can never make a community virtuous even in deed, let alone in thought. Divorce, neglect of marriage and prevention of offspring are crimes against society, against humanity and against God, and the only efficient prevention of these mischievous practices is in the becoming more imbued with the principles inculcated in the Scriptures of the Old and New Testaments, and this must be begun in the very early life of the child; even before the child goes to school; before it has learned the A. B. C., parents should begin to bring them up in the nurture and admonition of the Lord by reading to them the historical portions of the Bible, in short lessons and in short explanations, all the while aiming to impress upon the child's mind and heart the habit of receiving with implicit confidence and faith and affection every statement in the Holy Book as an assertion of the great God himself, without the most remote thought of calling in question one single word. Then will none want to go any farther than read the command "Multiply and replenish the earth," that there is no other sufficient cause of divorce than adultery and that whoever seeks to baffle God, to circumvent him in the operation of such physiological laws as he has enacted, will be found in the end to be ruining both body and soul for time and for eternity.

